# Cutaneous signs of selected cardiovascular disorders: A narrative review

**DOI:** 10.1515/med-2024-0897

**Published:** 2024-02-02

**Authors:** Marium Asif, Muhammad Hamza Yousuf, Usman Shakeel Farooqui, Abdulqadir J. Nashwan, Irfan Ullah

**Affiliations:** Faculty of Medicine, Dow University of Health Sciences, Karachi, Pakistan; Nursing Department, Hamad Medical Corporation, P.O. Box 3050, Doha, Qatar; Department of Internal Medicine, Khyber Teaching Hospital, Kabir Medical College, Gandhara University, Peshawar, Pakistan

**Keywords:** skin manifestations, cutaneous signs, cardiovascular diseases, diagnosis, treatment

## Abstract

Cardiovascular diseases are the leading cause of mortality and morbidity globally. Clinicians must know cutaneous signs of cardiovascular disease, including petechiae, macules, purpura, lentigines, and rashes. Although cutaneous manifestations of diseases like infectious endocarditis and acute rheumatic fever are well established, there is an indispensable need to evaluate other important cardiovascular diseases accompanied by cutaneous signs. Moreover, discussing the latest management strategies in this regard is equally imperative. This review discusses distinctive skin findings that help narrow the diagnosis of cardiovascular diseases and recommendations on appropriate treatment.

## 1 Introduction

Given that cardiovascular diseases impose a tremendous burden on individuals and governments, their appropriate diagnosis is crucial; their distinguishable cutaneous manifestations can serve as an enlightening tool for this objective. Skin is the largest organ of our body, acting as both a physical barrier and a home to various immune cells in innate immunity [1]. Various factors among different body organs cause identifiable lesions such as petechia, purpura, Janeway lesions, Osler’s nodes, subcutaneous nodules, erythema, lentigines, macules, xanthoma, digital cyanosis, and splinter hemorrhages. Furthermore, cutaneous manifestations are apparent in a wide range of cardiovascular disorders, such as infections, vasculitis, genetic disorders, metabolic disorders, and tumors. Dermatological lesions can present either due to the direct effect of the specific cardiovascular disease or as a part of cutaneous-cardiovascular conditions. In this review, we have discussed infectious, developmental, metabolic, and autoimmune causes involving both the heart and the skin. Skin lesions can act as significant clues in early diagnosis, differentiating diseases with similar presentation, preventing ensuing organ damage, determining rare genetic disorders, early detection of primary tumors, and timely treatment.

## 2 Acute cardiac diseases with infection or inflammation

### 2.1 Infective endocarditis (IE)

The life-threatening multisystem disease, IE, continues to pose significant clinical challenges, with an overall mortality of 30%. IE is an infection of the endocardial surface of the heart, mainly including heart valves, the mural endocardium, or a septal defect [2]. Several conditions and risk factors contribute to this condition, including calcific aortic stenosis, congenital heart diseases (ventricular septal defects, patent ductus arteriosus, and tetralogy of Fallot), previous endocarditis, valve intravenous drug use, prosthetic valves, mitral valve prolapse, diabetes mellitus, stroke, and healthcare-associated infections [3]. Native IE most usually affects the mitral valve, which is closely followed in decreasing order by the aortic valve, the combined mitral and aortic valve, the tricuspid valve, and in rare cases, the pulmonic valve. However, right-sided endocarditis is the predominant form in IV drug abusers. Infection rates for mechanical prosthetic and bioprosthetic valves are comparable [4]. Left-sided endocarditis causes complications through emboli formation and ensuing heart failure [3].

IE, occurring due to a combination of underlying valvular pathology and a transient bacteriemia, may present with cutaneous manifestations; hence, clinical diagnosis and management of IE require a dermatological examination. Many cutaneous findings are incorporated in the Duke’s criteria for IE; Janeway lesions, Osler’s nodes, purpura, and splinter hemorrhages act as essential clues in presumptive IE diagnosis [5]. Eight percent of patients in a study were found to have purpura and comparatively had larger cardiac vegetations [6]. Biopsy confirmed that purpuras observed in four patients were cutaneous leukocytic vasculitis [7]. More commonly, petechiae are seen above the clavicle, the mucosa of the palate, and the lids’ conjunctiva [8]. An association of higher extracardiac manifestations, particularly cerebral emboli, was observed in those with skin manifestation [6]. Osler’s nodes (seen more commonly in subacute IE) are associated with the bicuspid aortic valve. In addition, extracerebral emboli, particularly in the lungs and spleen, were significantly noted with Janeway lesions, occurring commonly in the acute form of the disease [5,6]. *Staphylococcus aureus* (SA), *Streptococcus viridans*, Groups A, C, and G streptococci, and enterococci are the most common pathogens responsible for IE [4]. SA is also the most common organism causing skin infections worldwide. From 1998 to 2009, an increase from 24 to 32% was observed in *Staph aureus*-related IE [3]. *Staph aureus*’s clumping factors A and B bind to the extracellular matrix, platelets, and fibrin in sterile vegetation, leading to inflammation, meanwhile evading host defenses through a self-produced biofilm [3,9]. Streptococci, another notorious cause for IE, also produces biofilm, thereby protecting itself from immune defenses.

Interestingly, dermatological lesions can guide clinicians to identify the causative organism, projecting and managing possible complications. A recent prospective multicenter study, with more than 1700 episodes of left-sided IE, compared the clinical behavior of the left-side IE caused by *Streptococcus agalactiae* (GBS) and SA. They revealed that SA left-sided IE presented significantly more cutaneous manifestation than GBS (32% for the former and 7.3% for the latter). Moreover, SA-caused IE got significantly complicated with more renal failure (45% vs 24%). Mortality was proportionally higher in the SA group (49% vs 29%), albeit not statistically significant [10].

### 2.2 Treatment

Treating IE requires antibiotics, particularly regimens of β-lactam antibiotics, aminoglycosides glycopeptide combined with an aminoglycoside. Moreover, SA-caused IE involves the use of daptomycin, rifampicin, linezolid, quinolones, and fosfomycin in combination with imipenem or daptomycin [10]. Despite preventing further destruction by clearing bacteremia, antibiotics cannot remove the necrotic tissue and bacterial vegetation; hence, further surgical interventions might be necessitated for some patients. Eventually, 15–20% of patients afflicted with IE will need surgery. There are a number of indications for surgical intervention, among which congestive heart failure (CHF) refractory to standard medical care is the primary indication. Mitral valvular damage is treated by valve repair, compared to aortic valve involvement in which root replacement and reconstruction become a requirement [9].

### 2.3 Acute rheumatic fever (ARF)

Group *Streptococcus pyogenes* (GAS), causing preceding pharyngitis or skin infections, can lead to a systemic inflammatory disease, ARF, occurring most commonly in children and manifesting with rheumatologic, cardiac, and dermatological signs and symptoms. The number of deaths through RF or RHD (rheumatic heart disease) is 300,000 per year globally and has decreased substantially in developed countries [11]. It primarily occurs in children living in low socioeconomic societies with overcrowding, bed-sharing, unhygienic lifestyle, poor dental health, and tobacco smoke exposure [12,13]. ARF occurs due to the phenomena of molecular mimicry in which T cells and antibodies act against a self-antigen, cardiac myosin in the myocardium and valves [13]. Diagnosis rests on a combination of clinical manifestations, such as fever, pancarditis, migratory polyarthritis, subcutaneous nodules, erythema marginatum, and Sydenham’s chorea. Carditis, as the most serious and lethal complication of ARF, is found in 80% of ARF cases and predominantly refers to valvulitis [13,14]. Carditis is diagnosed with progressive CHF, a new murmur, or pericarditis. Valve damage occurs due to T cell and Aschoff body infiltration. It results in regurgitation or stenosis of the valve, most commonly the isolated mitral valve followed by combined mitral and aortic valve involvement [14].

Children present with a febrile illness but can rapidly develop heart failure, necessitating prompt diagnosis with the help of cutaneous lesions. ARF’s major criteria, as noted by Jones in 1944, include skin findings such as subcutaneous nodules and erythema marginatum [12]. Cardiac valve involvement and deteriorating RHD can be caught early on by recognizing subcutaneous nodules, which occur commonly on periarticular sites and bony prominences [11]. Moreover, the rare presentation of erythema marginatum with Sydenham’s chorea without other physical symptoms can be diagnostic and immediate treatment can preclude initial carditis [15]. A unique association of Henoch palpable purpura (HPP) presented alongside ARF is observed, with ARF usually following HPP but can also precede it. Clinicians should be aware of this presentation as myocarditis in both ARF and HPP is worrisome due to its life-threatening nature [16].

### 2.4 Treatment

Since curing the inciting infection is entirely achievable, attention has been refocused on preventive strategies. RF can be properly managed through primary and secondary prevention, with primary being treatment of GAS infection and secondary preventing recurring bouts of RF, hence preventing its progression to RHD. The primary treatment goal is to eradicate streptococcal organisms. Benzathine penicillin, oral penicillin V, and amoxicillin are prescribed in accordance with various guidelines for primary prevention. Allergic individuals can be prescribed azithromycin, cephalexin, clindamycin, clarithromycin, and erythromycin. Secondary prevention requires 5–10 years of prophylaxis depending on the guideline and involves the use of benzathine-penicillin, oral penicillin V, erythromycin, and sulfonamide [12]. Corticosteroids (CS) should be preserved for severe carditis. While mild heart failure will respond to CS, digoxin might be needed in cases with severe heart failure. ARF occurs due to GAS infection in school-going children; therefore, developing a vaccine should be at the forefront to prevent the disastrous clinical manifestations [17,18].

### 2.5 Kawasaki disease (KD)

KD or Kawasaki syndrome, also known as mucocutaneous lymph node syndrome, is a systemic vasculitis of medium-sized arteries and unknown etiology, primarily affecting children from 1 to 5 years. Clinically presents as fever, edema of extremities, mainly over hands and feet, cervical lymphadenopathy, bilateral nonexudative conjunctivitis, and oral mucosal changes [19]. Dermatological manifestations include a polymorphous rash which is one of the requirements for diagnosis, while other cutaneous lesions can also be noted as in polymorphic exanthema (more than 90% of patients), erythema of the palms and sole, epidermal desquamation, and beau’s lines. Unique skin findings such as psoriatic eruption, atopic dermatitis, and erythema multiform can also present rarely [20]. Considering the disease’s predilection to coronary arteries, it can result in coronary artery aneurysms (CAA) and sudden death. Coronary arteries are almost always involved, and stenosis can result owing to arterial remodeling. Pericardium, myocardium, endocardium, and valves are also affected in the acute phase. Myocarditis causes a decrease in myocardial contractility and hence a decrease in cardiac output, causing increased mortality among patients [19]. Interestingly, a study revealed a correlation between increased coronary artery diameter and decreased nail fold capillary blood cell velocity, suggesting that dynamic capillaroscopy by Doppler flowmetry can elucidate early microcirculation abnormalities and be used for evaluating coronary artery status noninvasively [21].

### 2.6 Treatment

The principal goal of treatment is preventing CAA and other cardiac complications. KD for years has been treated with aspirin; however, it does not decrease the developing disorder in coronary arteries, and hence, IVIG is used in combination [19]. Treatment of IVIG reduces the rate of CAA from 25 to 3–5% [22]. Individuals unresponsive to initial IVIG can be given infliximab in place of the second infusion of IVIG. Anti-IL-1 receptor drug Anakinra is being studied in those unresponsive to traditional treatment [23]. Moreover, a mouse model showed that the IL-1 receptor antagonist blocked developing coronary abnormalities [24]. A recent mouse study has implicated Anakinra as being more effective against IL-1B in male mice than in females [25]. Steroids and pentoxifylline are rarely used as their effectiveness in initially treating KD has not been well established [19]. Results on the efficacy of a combination of IVIG treatment with CS on decreasing CAA are inconsistent [26,27].

## 3 Chronic cardiac diseases with inherited gene mutations

### 3.1 Noonan syndrome with multiple lentigines (NSML)

NSML, previously known as the LEOPARD syndrome, is included in a group of disorders called RASopathies. It is an extremely rare, autosomal dominant genetic disorder with only about 300 confirmed cases [28], resulting in abnormalities of multiple body systems, including the skin and cardiovascular system. PTPN11 gene mutation (encoding the protein tyrosine phosphatase SHP2) is the most common cause; other gene mutations include RAF1, BRAF, and MAP2K1 genes [29]. The main symptoms of NSML are multiple lentigines, cardiac abnormalities, growth retardation, distinct facial features including hypertelorism, sensorineural hearing loss, and genital anomalies like cryptorchidism and hypospadias [30]. Some case reports have also noted missing primary and permanent teeth [31]. However, this disorder has a wide range of clinical presentations with multiple additional symptoms. In some patients, it portrays dermatological symptoms of lentigines as the main feature, whereas other cases present severe cardiac symptoms and associated neuroblastoma, acute myeloid leukemia, acute lymphoblastic leukemia, or malignant melanoma [28,30]. Furthermore, this disorder has been associated with mild learning disabilities [30].

Cardiac symptoms usually present with electrocardiographic abnormalities because of conduction defects, pulmonary valve stenosis, and hypertrophic cardiomyopathy. Abnormalities in ECG can usually be noticed even if the patient has no cardiac structural defects, making it an important tool in confirming the diagnosis of NSML [30]. Hypertrophic cardiomyopathy is present in almost 70% of the cases [29], making it the most common cardiac defect. It usually involves the left ventricle, resulting in outflow obstruction and sudden death [30]. Nevertheless, it can also be clinically silent with only a murmur over the pericardium on physical examination [32]. A recent study has established a new echocardiographic association with a sigmoid septum and ventricular septal bugle, which may prove to be a helpful indicator in diagnosing NSML [33]. Frequent cardiovascular evaluations are critical since heart defects, especially cardiomyopathy, can progress with age and result in further complications. Multiple lentigines are the essential feature of this syndrome. These are numerous small black-to-brown macules, present usually on the face, neck, and upper torso [30] but can also appear over limbs, axilla, inguinal area, palms, soles, and external genitals [34]. Lentigines are usually noticed at 4–5 years of age and increase exponentially with age without any involvement of the mucosa [28,29,30]. Multiple lentigines are the most prominent diagnostic sign, highlighting the need for further tests to confirm the diagnosis. In 70–85% of cases where cardiac abnormalities are present at birth, the symptoms of the specific abnormality worsen after the development of multiple lentigines [35]. Consequently, lentigines can help identify the need for cardiovascular evaluation and immediate management of any worsening symptoms. Café-au-lait macules are also observed frequently and usually appear before lentigines [29,31]. According to one study, these light-to-dark brown macules were present in 80% of the cases [34]. In addition, NSML is linked to multiple melanocytic nevi, especially with RAF-1 mutation [34] and the potential of developing malignant melanoma [36]. Thus, dermatologists play an important role in identifying NSML as this disorder presents multiple cutaneous symptoms. Regular dermatoscopy follow-ups can also help early diagnose a potential melanoma [36].

### 3.2 Treatment

Since NSML involves multiple systems, treatment requires multidisciplinary care. Genital anomalies can be treated without any special interventions, hearing loss can be managed with hearing aids, or in some cases, cochlear implants [28,29]. Cryosurgery, laser treatment, tretinoin, and hydroquinone are a few treatment options for lentigines [28], but the most vital system for treatment is the cardiovascular system, especially in the case of hypertrophic cardiomyopathy. The treatment regimen involves beta-blockers and calcium channel blockers in patients with structural cardiac abnormalities and anti-arrhythmic drugs for life-threatening ventricular ectopics. One study suggested that the mTOR inhibitor rapamycin might be beneficial in reversing hypertrophic cardiomyopathy based on positive results in a mouse model [37]. However, another study on the human sample with PTPN11-mutated NSML reported different results from the animal studies, suggesting that caution should be taken when using rapamycin to treat heart defects in NSML [38]. Another recent study highlights that low-dose dasatinib may be an effective drug for treating hypertrophic cardiomyopathy, based on results in a mouse model [39]. Further studies should be conducted on human samples of NSML to confirm this new finding.

### 3.3 Cutis laxa (CL)

CL or elastolysis is an extremely rare and heterogeneous connective tissue disorder characterized by skin with no elasticity, producing a droopy appearance. The precise cause of CL is unknown; however, some mutations disrupting the normal functioning of elastic fibers are described, including a serine to proline amino acid substitution in the fibulin 5 gene or mutations in the elastin gene [40]. It also affects multiple body systems like cardiovascular, musculoskeletal, respiratory, and gastrointestinal systems [41]. CL has many types based on the mode of inheritance and whether the mutation is acquired or inherited. Different types of CL comprise acquired CL, X-linked recessive CL (also called occipital horn syndrome), autosomal dominant CL (ADCL), and autosomal recessive CL (ARCL). ARCL is divided into subtypes ARCL 1, ARCL 2, and ARCL 3 based on characteristic symptoms and the specific genes involved. These subtypes are then further classified into additional subtypes, including ARCL-1A, ARCL-1B, ARCL-1C (also called Urban-Rifkin-Davis syndrome), ARCL-2A, ARCL-2B, ARCL-3A (also called De Barsy syndrome A), and ARCL-3B (also called De Barsy syndrome B) [41].

CL presents with characteristic skin findings because of the dysfunctional connective tissue. The most apparent features are loose and wrinkled skin that bulges downwards due to the loss of normal skin elasticity around the face, extremities, and trunk, resulting in a prematurely aged appearance. Some types like ARCL are associated with early cutaneous involvements, while others like ADCL present cutaneous manifestations late in the disease course [40]. Hyperextensible and saggy skin is the main feature of multiple types of CL, including ARCL 1A, ARCL 1C, ADCL, and X-linked recessive CL [42]. ARCL 1B presents with similar dermatological findings but is usually not generalized and involves only certain areas where the skin folds like the axilla and groin [42]. In addition, ARCL2A/B shares the same gene mutation with wrinkly skin syndrome, resulting in excessive wrinkling of skin over the entire body [41,42]. ARCL 3 presents with thin, wrinkly, and translucent skin, due to which the underlying veins are visible in certain areas. Furthermore, abnormal hair cells are found specifically in X-linked recessive CL, resulting in fragile and twisted hair shafts [42]. Besides skin involvement, CL presents with a wide range of systemic involvement, such as digestive hernias, hollow viscus diverticula, genital prolapse, hypermobile joints and skeletal abnormalities, intellectual disability, and pulmonary emphysema [40,42]. The presence and severity of specific symptoms depend on the type of CL, with some types encompassing severe cardiac involvements and some not. ARCL 2 and ARCL 3 usually have mild or no cardiovascular manifestations. In contrast, ARCL 1 subtypes involve multiple cardiac symptoms. ARCL 1A is highly associated with the narrowing of pulmonary arteries and supravalvular AS [42,43], which, in turn, leads to the obstruction of the left ventricular (LV) outflow tract. ARCL 1B involves an exceptionally rare arterial condition characterized by distortion and twisting (tortuosity) of blood vessels. Abnormal elastic fibers in ARCL 1B can also cause aortic aneurysms [42,43]. Arterial tortuosity and aneurysms pose a high risk of fatal aortic rupture or dissections. In some cases, cardiac hypertrophy with bradycardia is also reported, especially with ARCL 1B variant [43]. ADCL, albeit manifesting cardiac or systematic complications rarely, is also linked to some cardiac involvement such as arterial aneurysms, aortic root dilatation, and bicuspid aortic valve [42,43]. Of note, the most serious complication of CL is considered the life-threatening cor pulmonale, resulting from progressive pulmonary emphysema, as well as aortic dissections.

### 3.4 Treatment

Dermatologists play an essential role in identifying cutaneous manifestations of CL and consulting with cardiologists for a complete cardiac workup to detect any possible anomalies at an early stage. There are no proper guidelines for treating CL, but symptomatic treatment can be followed to manage the patients. Plastic surgery can improve skin elasticity; nevertheless, the results are usually temporary as the skin symptoms occur again [40]. According to some case reports, arginine supplementation can improve cutaneous symptoms in ARCL 3B [42]. In the case of cardiac involvement, pharmacological interventions like beta-blockers can be used to stop the progression of aortic aneurysms and prevent the development of fatal aortic dissection [41]. Moreover, surgical intervention can be considered to resect the parts of aortic aneurysm.

### 3.5 Pseudoxanthoma elasticum (PXE)

PXE is a rare autosomal recessive genetic disorder, characterized by the mineral deposition in the connective tissue disrupting the normal functioning of elastic fibers (elastorrhexis). It is caused by mutations in the ABCC6 gene leading to a paucity of inorganic pyrophosphate, which functions mainly to prevent mineralization at unusual sites [44,45]. Thus, abnormal inorganic pyrophosphate results in mineralized elastic fibers mainly in the skin, the retina, and the cardiovascular system [44] but can also involve other body systems. Moreover, the question has been raised whether not only PXE patients but also heterozygous ABCC6 mutation carriers are at an increased risk of early cardiovascular complications [46].

In most cases, the first clinical evidence of PXE manifests as skin findings, usually in the form of small yellow-colored papules on the sides of the neck [44]. As the disease progresses, similar papules appear on areas where the skin folds like axilla, inguinal, and popliteal regions, and eventually, these papules combine into larger, elevated skin lesions [44]. In some cases, these skin lesions can also be associated with mucosal surfaces. Later on, the skin presents as loose and wrinkly in some areas, but not as severe as that observed in CL [44]. One cross-sectional study highlighted an association between the extent of skin lesions (determined by the number of affected skin sites) with the complications of PXE like eye and cardiovascular involvement [47]. Since the skin findings of these patients are usually asymptomatic, individuals with PXE are frequently not diagnosed until the presence of vascular or ocular sequelae [48]. Therefore, dermatologists play a key role in identifying the skin lesions at an early stage so that these patients can be appropriately managed before complications develop. Furthermore, localized variants have also been described where PXE is associated with the involvement of only one organ system [49].

PXE may result in visual impairment as the dysfunctional mineralization can involve the Bruch’s membrane in the choroid layer. This phenomenon can present as an ophthalmologic finding called “retinal angioid streaks” [44]. Further eye involvement can lead to hemorrhages and eventually blindness if left untreated. Moreover, PXE can lead to the mineralization of the elastic media and intima of the blood vessels. The disease mainly involves medium- and large-sized vessels. Coronary artery involvement leads to angina pectoris and acute myocardial infarction [44]. Furthermore, it can also lead to claudication and peripheral artery disease [44]. One study suggests that PXE is associated more with arteriosclerosis as a result of mineral deposition and, to a lesser degree, also involves atherosclerosis [45]. Renal artery involvement is rare and can lead to hypertension [50]. Due to the increased fragility of calcified submucosal vessels, PXE can cause vascular hemorrhages, mainly from the GI tract [51].

### 3.6 Treatment

There is no proper therapy for PXE, and therefore, the management is based on the presenting symptoms. In the case of ocular involvement, treatment with anti-vascular endothelial growth factor effectively prevents further complications and vision loss [44]. Diet, exercise, and smoking cessation are the bedrock of disease management, especially to reduce the extent of cardiovascular involvement. In addition, the consumption of some supplementary materials has been found to be beneficial. High blood pressure and serum lipids can exacerbate the disease course and therefore should be managed by lifestyle modification and medical therapy where necessary. One randomized trial highlighted the potential of magnesium oxide supplementation for improving the PXE-associated mineralization [52]. More trials are necessary to investigate this finding further. Another research article suggested the investigation of magnesium citrate specifically for treating PXE in future clinical studies [53]. A recently published letter mentioned significant improvement of PXE symptoms as a result of treatment with probenecid in a middle-aged woman [54]. Therefore, it is important to conduct clinical trials to confirm these potential treatments suggested by published articles to find an appropriate treatment for PXE.

## 4 Others

### 4.1 Lipid disorders

Lipid disorders incorporate a wide variety of metabolic disorders that cause abnormal blood lipid levels. The many causes of hyperlipidemia can be divided into primary and secondary subtypes. Primary hyperlipidemia is genetically inherited (including familial dyslipoproteinemia, familial hypertriglyceridemia, and familial lipoprotein lipase deficiency), whereas the secondary subtype results from unhealthy diets, hypothyroidism, medications (e.g., amiodarone, glucocorticoids), uncontrolled diabetes, and poor lifestyle regimen [55].

One common skin manifestation of lipid disorders is xanthoma. Xanthomas are benign lipid deposits localized within an organ system characterized by accumulations of lipid-laden macrophages. They are considered unique and essential clinical indicators of hyperlipidemia and hypercholesterolemia [56]. Some common types of xanthomas include plane, tendinous, tuberous, and eruptive xanthomas. Planar xanthomas have different types: xanthelasmas, diffuse plane xanthomas, intertriginous xanthomas, and palmar crease xanthomas [57].

Xanthelasmas are the most common type and present as soft, chamois-colored, or yellow-orange oblong plaques, usually near the inner canthi. Dyslipidemia is detected in approximately more than 50% of patients with xanthelasma. Childhood cases of xanthelasma are related to genetic lipid disorders such as familial hypercholesterolemia. Palmar crease xanthomas present as nodules and irregular yellowish plaques involving the palms and flexural surfaces of the fingers; they can be found in familial dysbetalipoproteinemia, along with multiple myeloma and primary biliary cirrhosis. Tuberous xanthomas present as yellowish or orange nodules involving the joints, particularly the elbows and knees, and are associated with familial dysbetalipidemia and familial hypercholesterolemia. Eruptive xanthomas are small yellow orange to reddish-brown papules that appear on the buttocks, extensor surfaces of the arms and thighs, knees, inguinal and axillary folds, and oral mucosa. They strongly suggest high triglyceride levels and are associated with chylomicronemia, familial dysbetalipidemia, and mixed dyslipidemia. Tendinous xanthomas are papules or nodules involving the tendons, particularly in the back of the hands, the dorsa of the feet, and the Achilles tendons. They are associated with familial hypercholesterolemia [58].

### 4.2 Treatment

The strong link between xanthomas and lipid disorders means that a diagnostic lipid profile is necessary for patients presenting with xanthomas. Xanthomas are not always related to underlying lipid disorders, but when they are, the mainstay of treatment depends on treating the underlying lipid disorders with lipid-lowering drugs and lifestyle changes to prevent atherosclerotic cardiovascular diseases. The efficacy of statins, fibrates, bile acid-binding resins, probucol, or nicotinic have been well documented for this matter. Moreover, different cosmetic treatments exist for xanthomas. For example, carbon dioxide lasers are the most common laser treatment used for xanthomas, offering excellent cosmetic results [59]. Similarly, many different cosmetic options exist for other types of xanthomas, and many recent studies have tried to further improve the available treatment options.

### 4.3 Cardiac myxomas

Cardiac myxomas are the most common primary tumors of the heart (40–50% of cardiac tumors), most commonly originating from the left atrium at the mitral annulus of the fossa ovalis border of the interatrial septum (IAS) [60]. Symptoms of atrial myxomas range from nonspecific constitutional signs to sudden cardiac death. Therefore, their diagnosis warrants a high index of suspicion. Primarily, symptoms can present because of left and right-sided heart failure, obstruction of the mitral valve, and systemic or pulmonary embolization. The most common symptom is dyspnea on exertion due to left-sided heart failure (75%), and other presenting symptoms consist of palpitations, syncope, pedal edema, chest pain (due to coronary embolization), and constitutional symptoms such as malaise, anorexia, fever, arthralgia, and weight loss [61]. Moreover, between 3.2 and 46.4% of cases are asymptomatic, and they only manifest symptoms when the tumor prolapses into the heart valves, obstructing the valve orifice [62].

Cutaneous lesions occur because of the systemic embolization of myxoma and include erythematous macules and papules (mostly acral), digital cyanosis, splinter hemorrhages, telangiectasia, livedo reticularis, Raynaud’s phenomenon, ulcerating lesions, reddish-violet malar flush, and violaceous, annular, and serpiginous lesions [63]. Moreover, some of these skin symptoms develop due to myoxomal secretion of interleukin-6, causing autoimmune symptoms like Raynaud’s phenomenon, malar erythema, and vasculitis [64].

Lastly, cardiac myxomas can be a part of a rare, autosomal dominant syndrome called “Carney syndrome”. The disease involves endocrinopathies, multiple endocrine and nonendocrine neoplasias, and lentiginosis. Carney syndrome is caused by defects in more than one gene, including the PRKAR1A gene encoding the regulatory subunit type 1α of protein kinase, abnormalities in the short arm of chromosome 2 (Carney), and chromosome 12 (Ki-ras oncogene). This disorder is characterized by cutaneous and mucosal pigmentation, myomas of the heart, endocrine, and nonendocrine neoplasms (breast, skin, and others) [65]. Cutaneous manifestations of the Carney complex include skin pigmentation (lentigines), cutaneous and mucosal myxomas, and melanocytes-derived tumors like epithelioid blue nevi and pigmented epithelioid melanocytoma [66]. These cutaneous lesions are not only the earliest and most common signs of Carney Complex but also include three of the major diagnostic criteria [65]. Echocardiography is the investigation of choice to define cardiac involvement. A recent clinical trial on patients with carney syndrome revealed that 42.6% of patients developed a cardiac tumor which was more than previously published studies. Hence, the importance of understanding the early cutaneous symptoms of carney syndrome as a potential clue to developing cardiac myxomas [67].

### 4.4 Treatment

Cardiac myxomas can be diagnosed with a transthoracic echocardiogram and transesophageal echo with very high sensitivity, and surgical excision is the treatment of choice [62]. Conventional treatment is performed via median sternotomy; however, mini thoracotomy with robotically assisted surgery and total endoscopic robotic resection have shown favorable outcomes [68,69]. Early treatment of cardiac myxomas is necessary for a better prognosis; therefore, dermatologists must understand the cutaneous manifestations of this disease, as they can sometimes be the initial symptoms [64].

### 4.5 Cardiac amyloidosis

Amyloidosis is a disease characterized by the deposition of beta-sheet fibrillar protein aggregates in various tissues; the deposition can be localized or involve multiple organs, such as the liver, spleen, kidneys, heart, nerves, and blood vessels [70]. The main forms of amyloidosis affecting the heart include amyloid light-chain (AL) amyloidosis and transthyretin-related amyloidosis [71]. Cardiac amyloidosis causes an increase in the thickness of the cardiac wall, leading to early death due to CHF. CHF can present with shortness of breath and swelling of the legs and abdomen, while amyloid deposition in the coronary arteries can cause angina [71]. Other cardiac manifestations include postural hypotension, sudden cardiac death, pericardial effusion, cardiac tamponade (rare), heart blocks, and cor pulmonale [71,72,73].

Skin manifestations commonly arise when amyloid infiltrates blood vessel walls and weakens them, resulting in intracutaneous hemorrhages such as purpura, petechiae, and ecchymosis. Moreover, increased abdominal pressure (due to coughing, sneezing, the Valsalva maneuver, and proctoscopy procedures) can cause periorbital purpura with high diagnostic specificity. Furthermore, subcutaneous nodules and plaques may be seen on the flexor surfaces, the face, and the buccal cavity, alongside the path of blood vessels. These signs are due to direct skin infiltration and can also present as scleroderma on the face, hands, and feet [74].

The diagnosis of cardiac amyloidosis is often delayed as the symptoms are usually nonspecific. When symptoms of CHF appear, a two-dimensional trans-thoracic echocardiography is performed. Most commonly, the echocardiogram will demonstrate LV thickening, which in the absence of hypertension, is highly suggestive of infiltrative cardiac diseases (not specific for amyloidosis). Other surfaces of the cardiac cavity can also thicken in this condition, resulting in right ventricular hypertrophy, thickened IAS, and thickened atrioventricular valves. The diagnosis of cardiac amyloidosis should strongly be considered when low-voltage complexes on ECG occur with evidence of thickened walls on an echocardiogram [71].

Skin symptoms such as purpura and ecchymosis, along with symptoms of cardiac failure, can help clinicians diagnose cardiac amyloidosis, especially when other nonspecific symptoms complicate or delay the diagnosis. Skin symptoms may occur in up to 40% of patients with AL amyloidosis. Most importantly, cutaneous signs are sometimes the early presenting symptom of the disease, and even in some cases, the only symptom before organ failure happens later in the disease [74]. Hence, it is extremely critical that dermatologists and cardiologists perceive these skin manifestations timely.

### 4.6 Diagonal earlobe crease (Frank’s sign)

A diagonal earlobe crease (Frank’s sign) is a wrinkle that extends 45° backward from the tragus to the auricle [75], and according to many clinical studies, it has a significant association with coronary artery disease (CAD) [76,77].

A recent systemic review, carried out to understand the diagnostic accuracy of diagonal earlobe crease for acute and chronic coronary disease, stated that although the diagnostic accuracy of earlobe crease is insufficient, because of its feasibility and accessible interpretation, it could be incorporated in the routine physical examination [78] (Table 1).

**Table 1:** Summary table

Disease	Etiology	Skin manifestations	Treatment
IE	Valvular pathology and transient bacteraemia	Janeway lesions Osler’s nodes Petechiae and purpura Splinter haemorrhages	Antibiotics Surgery
ARF	Molecular mimicry after GAS pharyngitis or skin infection	Subcutaneous nodules Erythema marginatum	Antibiotics
KD	Unknown	Polymorphous rash Erythema of palms and soles Epidermal desquamation Beau’s lines Unique findings as in psoriatic eruption, atopic dermatitis, and erythema multiforme occur rarely [20]	Aspirin and IVIG [19]
NSML	Autosomal dominant genetic disorder	Lentigines Café-au-lait macules Melanocytic nevi (especially with RAF-1 mutation) [34]	Cryosurgery, laser treatment, tretinoin, and hydroquinone for lentigines [28] Multidisciplinary care for the associated findings
CL	Mostly unknown (with some known genetic mutations)	Loose and wrinkled skin	Symptomatic treatment
Pseudoxanthoma elasticum	Autosomal recessive genetic disorder	Small, yellow-colored papules eventually combining into large, elevated skin lesions [44] Loose and wrinkled skin [44]	Symptomatic treatment
Lipid disorders	**Primary**: Genetic **Secondary**: Unhealthy diet and lifestyle, medications (e.g. amiodarone, glucocorticoids), and uncontrolled diabetes	Xanthoma Planar: Xanthelasmas (most common), diffuse plane, intertriginous, and palmar crease Tendinous Tuberous Eruptive [57]	Treating the underlying lipid disorder Carbon dioxide lasers for xanthoma [59]
Cardiac myxomas	Idiopathic	Erythematous macules and papules (mostly acral) Digital cyanosis Splinter haemorrhages Telangiectasia Livedo reticularis Raynaud’s phenomenon Ulcerating lesions Reddish-violet malar flush [63]	Surgical resection [68]
Cardiac amyloidosis	AL amyloidosis (plasma cell disorders), AA (chronic inflammatory diseases), Hereditary amyloidosis (genetic), Age related amyloidosis, dialysis-related (beta2-microglobulin) amyloidosis [79]	Intracutaneous haemorrhages (purpura, petechiae, and ecchymosis) Periorbital purpura (high diagnostic specificity) Subcutaneous nodules and plaques (flexor surfaces, face, buccal cavity, path of blood vessels)	Treating the underlying disease
CAD	Atherosclerosis [80]	Diagonal earlobe crease (Frank’s sign) [75]	Treating the underlying CAD

## 5 Conclusion

This review comprehensively gathered and discussed the characteristic dermatological lesions of cardiovascular disorders, which can benefit both cardiologists and dermatologists. The presence of cutaneous lesions in IE can help distinguish between the underlying pathogen and therefore allow proper prevention of life-threatening complications. Moreover, cutaneous symptoms in rare genetic diseases such as NSML can guide the diagnosis and highlight the importance of cardiovascular evaluation in these patients, leading to early detection of cardiac anomalies such as hypertrophic cardiomyopathy. Similarly, when noting loose and saggy skin, physicians can be vigilant for detecting aortic aneurysms in CL. Skin lesions and their characteristic pattern of progression are usually the initial clinical manifestation of PXE and hence confirm the diagnosis before the development of cardiovascular manifestations. Cardiac myxomas can be fatal, but dermatological manifestations can facilitate early diagnosis and preclude subsequent mortality. The importance of identifying cutaneous lesions is not limited to dermatologists, rather encompasses a variety of medical fields such as general physicians, pediatricians, and cardiologists.
